# JNER at 15 years: analysis of the state of neuroengineering and rehabilitation

**DOI:** 10.1186/s12984-019-0610-0

**Published:** 2019-10-30

**Authors:** David J. Reinkensmeyer

**Affiliations:** 1 Department of Mechanical and Aerospace Engineering, University of California at Irvine, California, USA; 2 Department of Anatomy and Neurobiology, University of California at Irvine, California, USA; 3 Department of Biomedical Engineering, University of California at Irvine, California, USA; 4 Department of Physical Medicine and Rehabilitation, University of California at Irvine, California, USA

**Keywords:** Neuroengineering, Rehabilitation, Movement, Neuroscience, Wearable, Robotics

## Abstract

On JNER’s 15th anniversary, this editorial analyzes the state of the field of neuroengineering and rehabilitation. I first discuss some ways that the nature of neurorehabilitation research has evolved in the past 15 years based on my perspective as editor-in-chief of JNER and a researcher in the field. I highlight increasing reliance on advanced technologies, improved rigor and openness of research, and three, related, new paradigms – wearable devices, the Cybathlon competition, and human augmentation studies – indicators that neurorehabilitation is squarely in the age of wearability. Then, I briefly speculate on how the field might make progress going forward, highlighting the need for new models of training and learning driven by big data, better personalization and targeting, and an increase in the quantity and quality of usability and uptake studies to improve translation.

## Background

With JNER turning 15, and having just finished 5 years as the second Editor-in-Chief of JNER, it seems appropriate to reflect on where the field has been and where it is headed. The purpose of this editorial, therefore, is to briefly analyze the current status and future prospects of the field of neuroengineering and rehabilitation through the lens of the papers published at JNER, as well as my own experiences in the field. In particular, I ask – “How is the nature of neurorehabilitation research evolving?” and “How might the field progress over the next 15 years?”

## Main text

### Increasing use of Technology in Neurorehabilitation

**Fig. 1:**
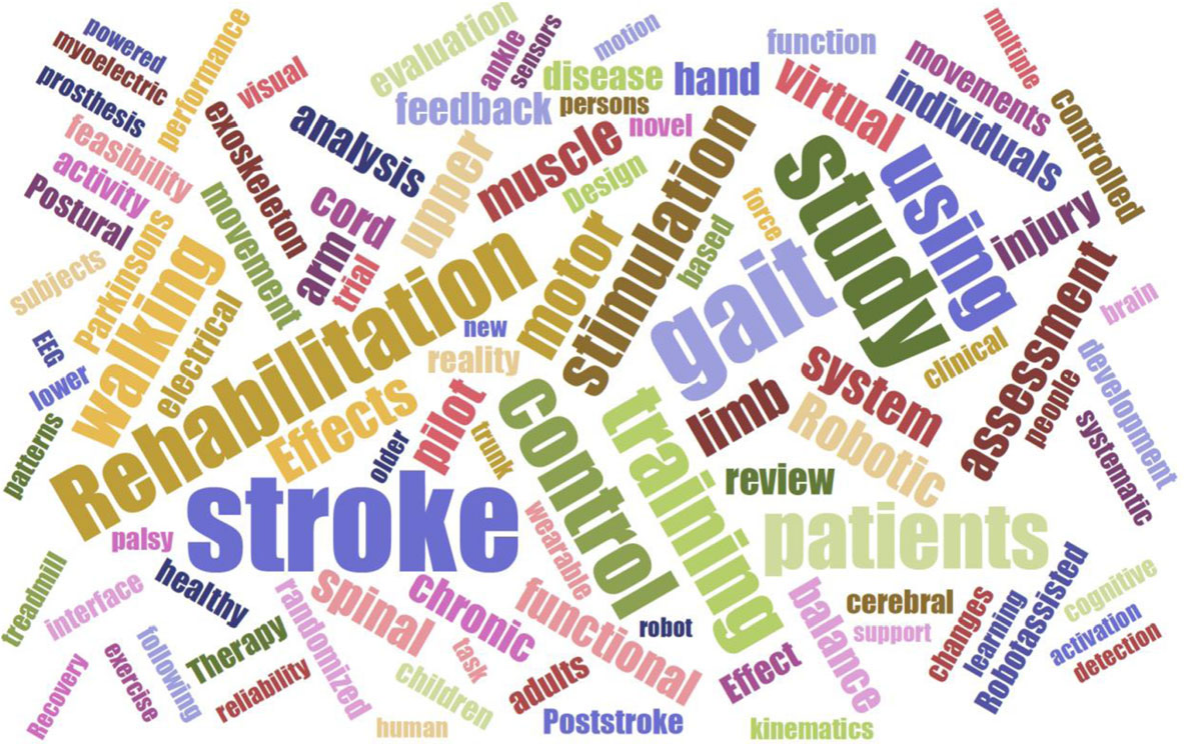
Word cloud generated from the titles of 1231 papers published in JNER over the past 15 years. One sees the integration of clinical and technological terminology. Generated at https://www.jasondavies.com/wordcloud/

In the last 15 years, an important way that neurorehabilitation research has evolved is that it has become increasingly familiar with and reliant on advanced neuroscience and engineering technologies. The trajectory of JNER is evidence of this integration. JNER was founded in 2004 as a “forum to discuss how neuroscience and biomedical engineering are reshaping physical medicine and rehabilitation” [1]. Figure 1 shows a word cloud generated from the titles of all papers published in JNER since its founding. One sees a robust mixture of clinical and technological terminology, particularly focused on applications in movement control and training after stroke, spinal cord injury, Parkinson’s disease, and amputation; the broader field of rehabilitation has also seen this integration [2].

**Fig. 2:**
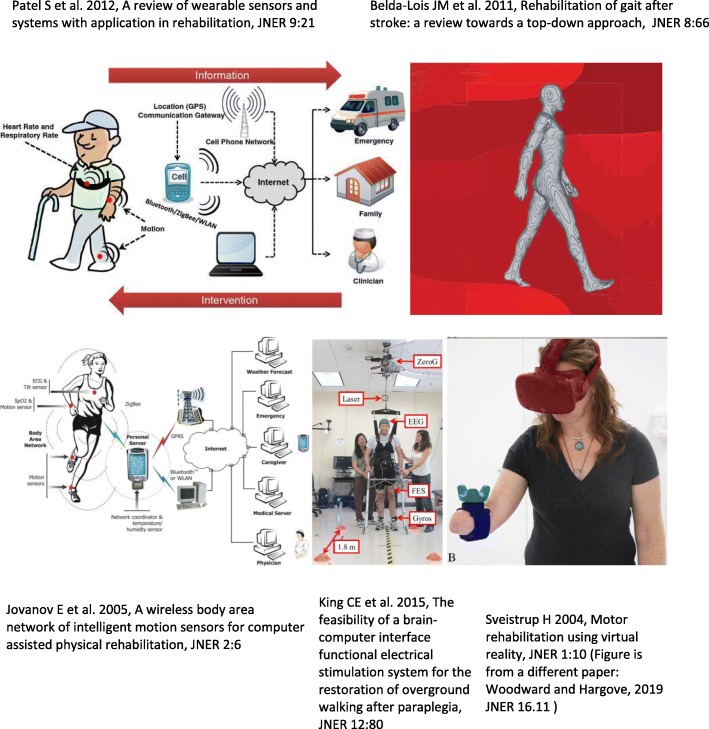
Representative figures from the most accessed papers of JNER [3, 4]. The virtual reality figure is from [5]. These highly accessed papers highlight the strong public interest in the blending of technology with rehabilitation

One also sees this blending of technology and clinical neurorehabilitation science in what are currently the top five accessed papers over the history of the journal (Fig. 2). The most accessed paper (243 K accesses) is a seminal review on wearable sensors in rehabilitation, which expertly categorized key enabling technologies and defined the major areas of application of wearable technology [3]. The second most-accessed paper (112 K accesses) is a review of gait training after stroke that systematically considers various technological approaches including functional electrical stimulation, robotics, and brain-computer interfaces [6]. The third most-accessed paper (111 K accesses) demonstrated one of the first wireless body area networks for rehabilitation [7]. The fourth most-accessed paper (96 K accesses) was the first demonstration of a BCI-controlled functional electrical stimulation (FES) system for walking after spinal cord injury [8]. Numerous prominent news outlets reported on this first demonstration of direct brain-controlled FES walking, contributing to this paper to have the highest Altmetric score at JNER. Finally, the fifth most-accessed paper (70 K accesses) is an early and influential review on using virtual reality for motor rehabilitation [4]. These papers all present innovative concepts and results for enhancing rehabilitation with technology, rather than analysis of routine clinical use of new technologies in rehabilitation, highlighting the prominent role that JNER has played in publishing new conceptual work.

**Fig. 3:**
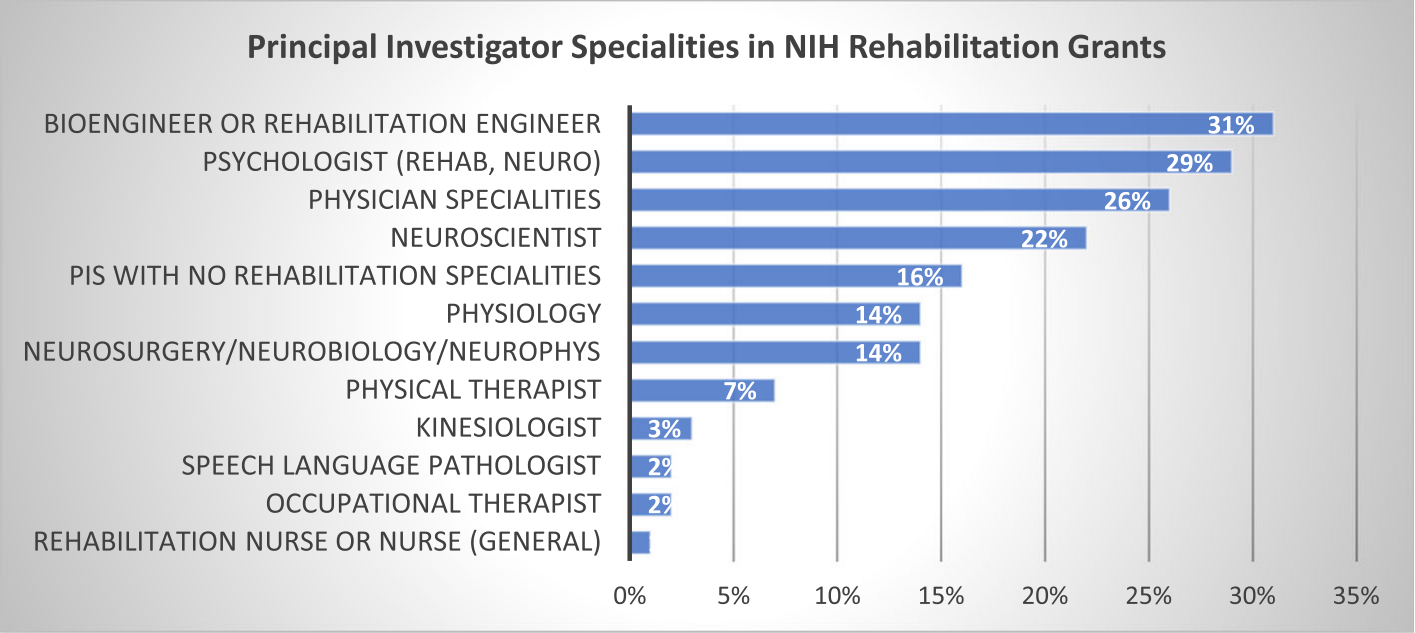
Evidence of the increasing role of technology in rehabilitation science. Shown are the frequency of specialties listed by principal investigators in rehabilitation grant application bisosketches. Percentages were calculated from 1178 applications (first submissions and first re-submissions) between 2007 and 2018. Data were shown at the December 3, 2018 National Advisory Board for Medical Rehabilitation Research meeting and were produced by NIH Office of Portfolio Analysis

Even though JNER takes a relatively specialized approach to rehabilitation research by studying how cutting-edge technology might reshape rehabilitation, JNER was the top ranked journal in the rehabilitation category in Scopus for 2017. For 2018 JNER was ranked second in the rehabilitation category in Scopus and fourth in the rehabilitation category in Web of Science. Relatedly, in the U.S., the National Institutes of Health has also seen a rise in the prominence of rehabilitation research that incorporates advanced engineering tools. An analysis done in 2018 by NIH analysts found that “bioengineer or rehabilitation engineer” was the most frequently listed specialty by lead PI in the 1117 funded grant applications in the rehabilitation domain between 2007 and 2018 (Fig. 3).

In my personal observations, I have been impressed as I circulated at poster sessions at recent neurorehabilitation and neuroscience meetings. Physical and occupational therapist researchers now fluently and routinely use cutting-edge technologies – including robotics, brain monitoring and stimulation, and sophisticated data analysis techniques – to aid their research. Use of cutting-edge technologies for research by therapists was uncommon 30 years ago. In 2015, the Eugene Michels Lecture at the American Physical Therapy Association Combined Sections Meeting was titled “Rehabilitation technology: Friend or Foe?”. Then, in 2019, the American Physical Therapy Association Combined Sections Meeting hosted an educational debate: “Why we love AND hate our robots.” [5] And yet, technology use in neurorehabilitation research is at present much more prevalent than technology use in neurorehabilitation practice, an issue of translation discussed further below.

**Fig. 4:**
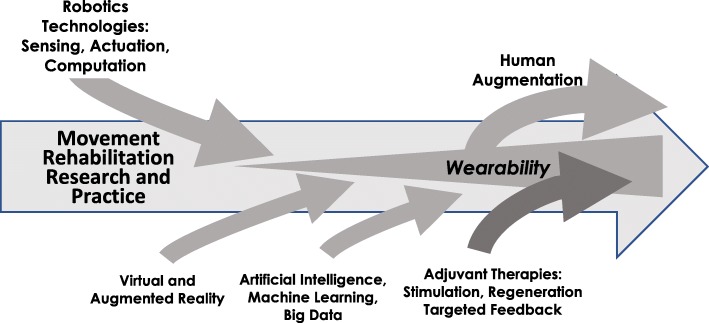
Conceptual timeline of the integration of various technologies in movement rehabilitation research and practice. The current state of the field is characterized by increased use of artificial intelligence, generation of big data, and experimentation with adjuvant therapies. We are squarely in the age of wearability, and wearable devices for rehabilitation are spinning out to applications in human augmentation

Figure 4 shows a conceptual timeline for how various technologies have entered movement rehabilitation research (and, at a slower rate, rehabilitation practice). The robotics revolution began in the late 1980s/early 1990s (where I define “robotics” broadly, meaning sensing, actuation, and computational technologies). Virtual reality in neurorehabilitation increased soon after. Now, we are seeing an increased incorporation of artificial intelligence, and increased experimentation with adjuvant therapies (such as brain stimulation and targeted feedback). Additionally, as I discuss below, we are squarely in the age of wearability.

This integration of technology and neurorehabilitation has several benefits spanning scientific discovery, technology development, and clinical practice, including:

1. Better science: New technologies allow better rehabilitation science to be done because they increase the reliability of measurements, quantify things not previously measured (e.g. wearable sensing of daily activity), and allow entirely new techniques to be applied in rehabilitation (e.g. robotic error augmentation).
2. Improved technology development: The emergence of cross-disciplinary research teams that include clinicians, users, and engineers allows crucial multi-directional information flow. Greater familiarity with clinical rehabilitation problems helps engineers better design new technologies, and greater familiarity with technology development and translation processes helps clinicians and users better communicate their vision, needs, and desires.
3. Potentially better practice: Although uptake of new technologies in routine clinical practice is slower than uptake into research, most rehabilitation facilities are now experimenting with new technologies, and, in some cases, are finding ways to enhance their practice.

Of course, there are possible disadvantages to overly focusing on technology in rehabilitation research, including a narrowing of perspective, increasing cost and complexity, and discounting of important human factors. When I was beginning as a post-doctoral fellow at the Rehabilitation Institute of Chicago in 1994, I met with renowned prosthetics and assistive technology researcher Dudley Childress to discuss my goal of building a robotic device for rehabilitation therapy. He asked me, “But what about the human touch?” Understanding how rehabilitation technology can best complement the human touch – and can make all of us more human – remains a key goal. It is significant that “psychologist” (including rehabilitation psychologist and neuropsychologist) was the second most frequently listed specialty by lead PI at NIH for rehabilitation in Fig. 3.

### Improved rigor of and access to research

Another way the field of rehabilitation has continued to evolve is by improving the rigor of research. A key moment was in 2014 when a consortium of rehabilitation journals began requiring the “complete and transparent reporting of research and methods” using established reporting guideline checklists [9]. NIH has promoted improved research and clinical trial rigor in response to the reproducibility crisis [10]. At the level of personal observation, I have seen a trend toward larger sample sizes in studies with human participants. To check my subjective impressions, I compared the first 10 experimental papers published in JNER versus the last 10 experimental papers: the average sample size was 11 compared to 48. I expect to see this trend continue as the quality of rehabilitation science increases.

Access to research results is also improving. When they founded JNER, Paolo Bonato and Biomed Central were at the vanguard of the open access movement. The NIH Public Access Policy was drafted in 2004, JNER’s first year, and mandated in 2008. NIH and other funding agencies across many countries now require the research they fund to be made available to the public for free within 1 year of publication. Since JNER provides immediate, open access, any interested person around the world can read the latest research. This is particularly important for rehabilitation, because this means that inventors, persons with a disability (who may themselves be inventors as well as consumers), and rehabilitation therapists and caregivers worldwide can all interact with the latest findings. The power of the open access movement should not be underestimated – surely a larger, broader, more diverse readership will ultimately produce more innovation and greater uptake of promising methods of rehabilitation.

### Innovative research paradigms

**Fig. 5:**
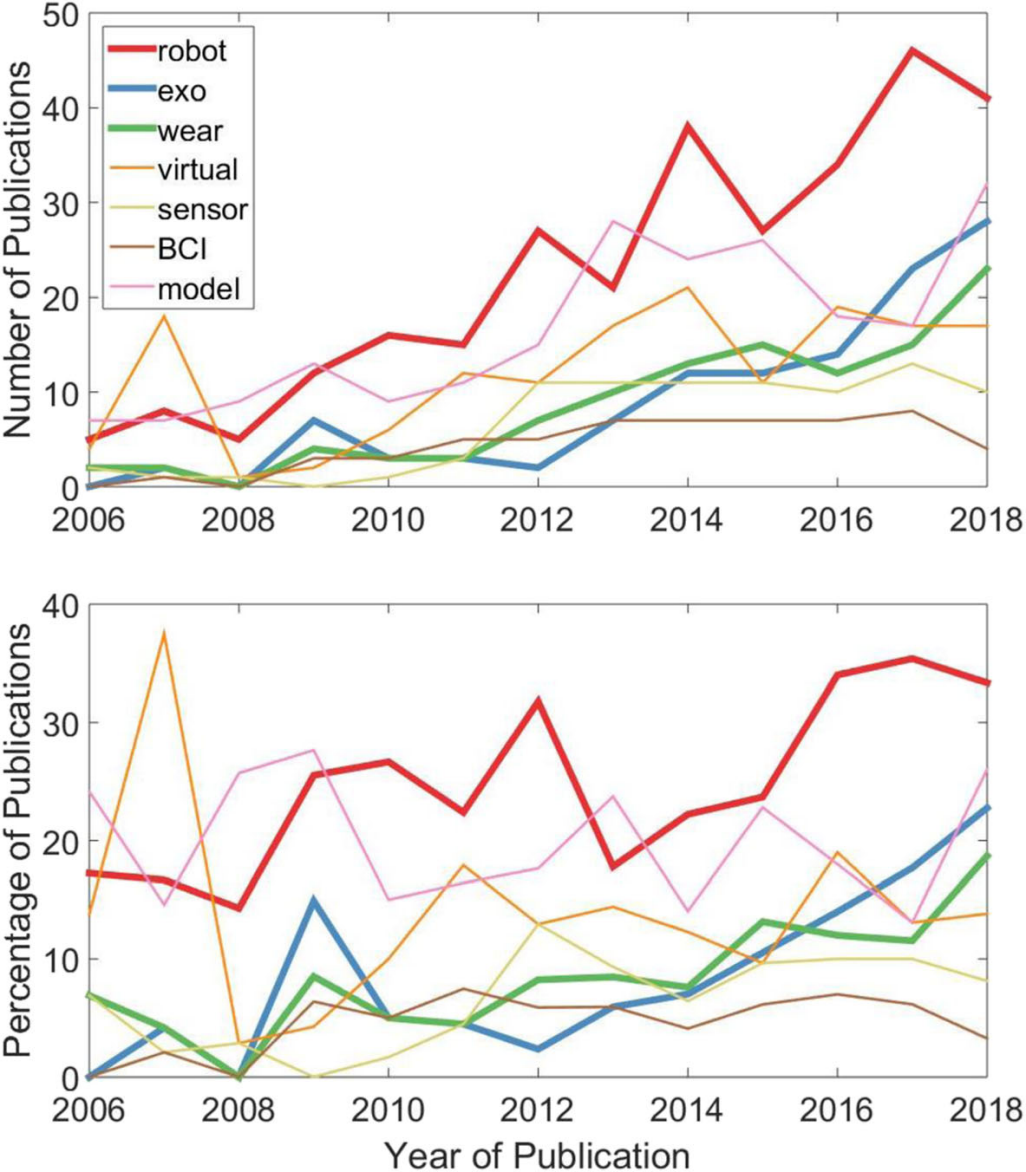
Analysis of the title, abstracts, and key words of the 1231 papers published in JNER over the past 15 years for the number (top) and percent (bottom) of papers that use the words robot*, exo*, wear*, virtual, sensor, brain-computer, and model. The fastest growing terms over the past 5 years are “exo*”, “wear*” and “robot*”

I also want to highlight an evolution in the types of technical topics being published at JNER. I analyzed the title, abstracts, and key words of the 1231 papers published in JNER over the past 15 years for the percent of papers that use the words robot*, exo*, wear*, virtual, sensor, brain-computer, and model. The fastest growing term over the past 5 years is “exo*”, followed by “wear*” and “robot*” (Fig. 5). Much of this work has to do with mechanically assistive devices worn on the limbs, but also includes many studies on wearable sensors, including phones and watch-like devices. This analysis suggests that we are in the “wearable” age of neurorehabilitation technology development (Fig. 4).

Analysis of the ten most cited JNER papers published in 2017 supports this idea (Table 1). Eight of the papers deal with devices worn in some way (1 exoskeleton paper, 3 prosthetic papers using EMG/EEG/or nerve-signal based control, 2 wearable sensor papers, and 2 papers using surface or implanted stimulation). The two “non-wearable” papers focused on virtual reality for rehabilitation (monitored using EEG – arguably a wearable system), and a systematic review of robotic gait rehabilitation (with the Lokomat, which, arguably, also is “worn” although not portable). Interestingly, five of these most-cited papers shown in Table 1 also make use of advanced machine learning techniques, which brings to mind a quote from Kevin Kelly, a co-founder of Wired magazine: “I think the formula for the next 10,000 start-ups is to take something that already exists and add AI to it.” [21] In neurorehabilitation, AI will play a key role in translating wearable technologies so that they can be used by diverse people in diverse settings. And finally, three of these most cited papers consider synergies between brain stimulation or monitoring technology and rehabilitation. The idea that adding an adjuvant treatment can improve conventional rehabilitation therapy is a major stream of research activity now entering movement rehabilitation as depicted in Fig. 4.

**Table 1:** The ten most-cited papers published in JNER in 2017 (from Web of Science, accessed 9-1-2019). W = paper involves a technology worn (or implanted). AI = paper involves techniques from artificial intelligence, such as pattern recognition or machine learning. S = paper involves synergistic application of an adjuvant technique to understand or enhance therapy

Li et al. 2017, A motion-classification strategy based on sEMG-EEG signal combination for upper-limb amputees, JNER 14:2 [11] (34 cites)	W, AI
Wang et al. 2017, Interactive wearable systems for upper body rehabilitation: a systematic review, JNER 14:20 [12] (29 cites)	W, AI
Wendelken et al. 2017, Restoration of motor control and proprioceptive and cutaneous sensation in humans with prior upper-limb amputation via multiple Utah Slanted Electrode Arrays (USEAs) implanted in residual peripheral arm nerves JNER 14:121 [13] (24 cites)	W, AI
Calabro RS et al. 2017, The role of virtual reality in improving motor performance as revealed by EEG: a randomized clinical trial, JNER 14:53 [14] (22 cites)	(W), S
Galle et al. 2017, Reducing the metabolic cost of walking with an ankle exoskeleton: interaction between actuation timing and power, JNER 14:35 [15] (21 cites)	W
Parastarfeizabadi and Kouzani 2017, Advances in closed-loop deep brain stimulation devices, JNER 14:79 [16] (21 cites)	W, S
Nam KY et al. 2017, Robot-assisted gait training (Lokomat) improves walking function and activity in people with spinal cord injury: a systematic review, JNER 14:24 [17] (19 cites)	(W)
Elsner B et al. 2017, Transcranial direct current stimulation (tDCS) for improving capacity in activities and arm function after stroke: a network meta-analysis of randomised controlled trials, JNER 14:95 [18] (13 cites)	W, S
Dellacasa Bellingegni et al. 2017, A1 NLR, MLP, SVM, and LDA: a comparative analysis on EMG data from people with trans-radial amputation, JNER 14:82 [19] (13 cites)	W, AI
Nguyen H et al. 2017, Auto detection and segmentation of daily living activities during a Timed Up and Go task in people with Parkinson’s disease using multiple inertial sensors, JNER 14.26 [20] (13 cites)	W, AI

Wearable rehabilitation will continue to mature, and indeed represents a new paradigm in rehabilitation. It seems to me that much wearable robotic device work focused initially on the goal of providing assistance to accomplish a task (usually walking), a difficult and important goal. Indeed, adding actuation to orthotics has begun to improve everyday mobility. However, wearable robotic device work has also quickly became interested in the therapeutic effects of assistance. Until now, rehabilitation therapy has been something mainly done by going to a facility. With AI-enhanced, wearable devices, some aspects of rehabilitation therapy will become continuously accessible. The transition will be like what has happened with knowledge seeking – instead of going to a facility (like a library, bookstore, or school), one now frequently learns from information provided by mobile devices that we carry with us. In the same way, we will learn to move with assistance (physical and informational) provided by wearable devices. We will essentially be able to take a sort of “avatar” of our rehabilitation therapist with us as we move at home or in the community, thereby improving our access to rehabilitation therapy no matter where we live.

The rise of wearables is evident in a second innovation – the Cybathlon competition, which was first held in 2016. As described in an editorial in JNER, Robert Riener conceived of the Cybathlon as a new sort of Olympics, in which a pilot with a disability (such as paralysis or an amputation) collaborates with a technical team to race an assistive technology (such as an FES bicycle or wheelchair) or worn device (such as a powered prosthetic arm or leg, a BCI, or a legged exoskeleton) [22]. As can be seen in a fascinating collection of papers published in JNER [23–30], this approach combined neuroscience, engineering, and rehabilitation with very strong usability and reliability constraints in a way not seen before. Because of the Cybathlon, more people are now working on diversely, inventive ways to solve truly stubborn problems in rehabilitation – including stair climbing wheelchairs, agile powered leg exoskeletons, robustly responsive brain-computer interfaces, and dexterous prostheses. By framing technology development as a public, international competition, Cybathlon has promoted a new approach to assistive technology research. There are some limitations to this approach, for example, in emphasizing task completion and speed over quality of movement, ease-of-use, or long-term comfort, although the next event will provide secondary medals for ergonomics. Nevertheless, I expect Cybathlon to continue to increase the number of studies that systematically optimize the use of specific technology by a specific user over an extended period of time. This type of study will help make assistive technologies more individualized, more accessible, safer, and more hardened so that they perform well in demanding circumstances.

### Human augmentation

I have also been excited to see cutting-edge papers submitted to JNER on new approaches to human augmentation. JNER published the first study that demonstrated a metabolic reduction in walking of non-impaired persons using an autonomous exoskeleton [31] and continues to publish fascinating work in this field (e.g. [15, 32–38]). Look for a forthcoming review that tracks the continuing improvements in metabolic reduction that have been achieved with various types of exoskeletons. A recent special issue on tDCS includes a review on the application of brain stimulation technology to enhance athletic performance [39]. Associate Editor Lou Awad, an emerging leader in the human augmentation field, recently remarked: “If we ever need to change the name of JNER, I’d vote for JMAR -- Journal of Movement Augmentation and Rehabilitation.”

My impression of movement augmentation studies is that they are putting to the test our current understanding of biomechanics and neural control in new ways. They are also bringing together a community of people with and without disabilities around a common goal – enhancing movement. This is similar to the universal design movement, which focuses on design of the built world to benefit both people with and without a disability. The movement augmentation movement (a term that sounds especially dynamic!) focuses on design of the worn world to benefit both people with and without a disability. Just as curb cuts spun out of universal design into general use, movement augmentation devices are spinning out of neuroengineering and rehabilitation research for general use (Fig. 3). Look for continued beneficial interactions between augmentation and rehabilitation. I’d like JNER to be a premiere venue for movement augmentation studies, and will continue to welcome submissions in this area.

## Conclusions

In light of this evolution, how might the field make progress going forward? I suggest that we need new models of training and learning driven by big data, better personalization and targeting (i.e. precision medicine), and more usability and uptake studies to improve translation.

There are still tremendous opportunities to better inform rehabilitation techniques with neuroscientific findings. For example, JNER recently published a collection of papers on brain stimulation as an adjuvant therapy to increase training-related plasticity [40]. The field of regenerative rehabilitation is emerging and will surely benefit from the application of rehabilitation technology [41, 42]. And, as my colleagues and I have argued in papers published in JNER, better computational models will be important for moving rehabilitation forward [43, 44]. JNER is a leader in modeling – 20% of papers published in JNER mention the world “model” in the title or abstract – yet few of these models deal with plasticity, and clinically useful models are yet to be developed. While 33% of the papers published at JNER mentioned “training”, only 10% mentioned learning and 5% plasticity.

There needs to be a critical mass of researchers focused on computational modeling of rehabilitation, identifying clinical problems where existing modeling technology can improve care. Expansion of models into new areas will require increased openness and support from key funding agencies. The technology revolution in rehabilitation is the great facilitator here, making the moment opportune: machines are helping to build the larger, finer resolution data sets needed to form and test models.

We need greater sophistication in the way a rehabilitation technology is matched to each person’s impairments and goals. I mentioned the Cybathlon and movement augmentation studies above as crucibles for this sort of work. Machine learning is also a powerful tool for selecting control algorithms or treatment approaches that work best for specific individuals. In robot-assisted and other technology-assisted rehabilitation approaches, it will be increasingly important to refine methods to identify who can benefit from what type of training and why (for a sample of recent interesting work in this area published at JNER, see: [45–48]). I expect that better targeting of robot-assisted therapy will at least double its clinical benefits. Precision medicine is a major theme at NIH (e.g. the All of Us Research Program). Neurorehabilitation technology research needs to follow this trajectory as well.

Translation remains a significant barrier limiting the implementation of new technologies in clinical practice. Users and their clinicians must be more intimately involved in teams working toward the development and testing of new technologies. Funding agencies can facilitate this by rewarding partnering of engineering and clinical rehabilitation institutions. The field needs to more rigorously document and understand the rehabilitation technology testing process by designing clinical usability studies that go beyond presenting survey results. Currently only 2.4% of the papers published in JNER use the term “usability” in the title, abstract, or keywords. It is critical to better understand what causes the slow uptake of new technologies, and to identify the best practices for incorporating new technologies into clinical practice [49]. Further, it is increasingly apparent that the benefits of rehabilitation technology depend on the psychology of the user, including motivation, effort, and success. Therefore, the fundamental design principles of rehabilitation technology must be grounded in the behavioral science and neurophysiological mechanisms of these psychological factors. Clearly, this goal, as well as the other goals I outlined above, will require increasing transdisciplinary teamwork.

Fifteen years ago I imagined what rehabilitation technology would look like if I had a stroke in 2036 [50]. I won’t be as specific here. But, here is what I hope for: more people with and without disabilities will more frequently take advantage of rehabilitation-inspired technologies because they are truly useful to them; sensor-based data, computational modeling, and artificial intelligence will increasingly enhance rehabilitation science with more statistical power, promoting better mechanistic understanding and better outcomes; and adjuvant therapies will help rehabilitation go beyond what it can currently achieve. I look forward to witnessing these developments through papers submitted to JNER.
